# Shrimp Serine Proteinase Homologues *Pm*MasSPH-1 and -2 Play a Role in the Activation of the Prophenoloxidase System

**DOI:** 10.1371/journal.pone.0121073

**Published:** 2015-03-24

**Authors:** Miti Jearaphunt, Piti Amparyup, Pakkakul Sangsuriya, Walaiporn Charoensapsri, Saengchan Senapin, Anchalee Tassanakajon

**Affiliations:** 1 Center of Excellence for Molecular Biology and Genomics of Shrimp, Department of Biochemistry, Faculty of Science, Chulalongkorn University, Bangkok, Thailand; 2 National Center for Genetic Engineering and Biotechnology (BIOTEC), National Science and Technology Development Agency (NSTDA), Klong 1, Klong Luang, Pathumthani, Thailand; 3 Center of Excellence for Shrimp Molecular Biology and Biotechnology (Centex Shrimp), Mahidol University, Bangkok, Thailand; Uppsala University, SWEDEN

## Abstract

Melanization mediated by the prophenoloxidase (proPO) activating system is a rapid immune response used by invertebrates against intruding pathogens. Several masquerade-like and serine proteinase homologues (SPHs) have been demonstrated to play an essential role in proPO activation in insects and crustaceans. In a previous study, we characterized the masquerade-like SPH, *Pm*MasSPH1, in the black tiger shrimp *Penaeus monodon* as a multifunctional immune protein based on its recognition and antimicrobial activity against the Gram-negative bacteria *Vibrio harveyi*. In the present study, we identify a novel SPH, known as *Pm*MasSPH2, composed of an N-terminal clip domain and a C-terminal SP-like domain that share high similarity to those of other insect and crustacean SPHs. We demonstrate that gene silencing of *Pm*MasSPH1 and *Pm*MasSPH2 significantly reduces PO activity, resulting in a high number of *V*. *harveyi* in the hemolymph. Interestingly, knockdown of *Pm*MasSPH1 suppressed not only its gene transcript but also other immune-related genes in the proPO system (e.g., *Pm*PPAE2) and antimicrobial peptides (e.g., *Penmon*PEN3, *Penmon*PEN5, crustin*Pm*1 and Crus-like*Pm*). The *Pm*MasSPH1 and *Pm*MasSPH2 also show binding activity to peptidoglycan (PGN) of Gram-positive bacteria. Using a yeast two-hybrid analysis and co-immunoprecipitation, we demonstrate that *Pm*MasSPH1 specifically interacted with the final proteinase of the proPO cascade, *Pm*PPAE2. Furthermore, the presence of both *Pm*MasSPH1 and *Pm*PPAE2 enhances PGN-induced PO activity *in vitro*. Taken together, these results suggest the importance of *Pm*MasSPHs in the activation of the shrimp proPO system.

## Introduction

Invertebrates primarily rely on innate immunity mediated by cellular and humoral responses to combat invading pathogens. A cellular immune response involves different types of blood cells that recognize conserved pathogen associated molecular patterns (PAMPs) and leads to activation of nodule formation, encapsulation, and phagocytosis of the microorganisms [1–3]. In contrast, the humoral defense involves production and secretion of several immune proteins such as antimicrobial peptides (AMPs), as well as proteinases and their inhibitors that associated with the clotting and melanization cascade in hemolymph [1–3]. Melanization is one of the most rapid and effective humoral responses that is driven by a key enzyme called phenoloxidase (PO) and is tightly regulated by the proPO activation cascade [4–6]. Upon recognition of microbial PAMPs, such as lipopolysaccharide (LPS) from Gram-negative bacteria, peptidoglycan (PGN) from Gram-positive bacteria or β-1,3-glucans from fungi, by pattern- or pathogen-recognition receptors (PRPs) [7], several serine proteinases generate the active PO required for melanin synthesis [4,8,9].

Several reports in insects have demonstrated the essential function of proPO in surviving microbial infection [10–13]. In crustaceans, it has also been demonstrated that the freshwater crayfish *Pacifastacus leniusculus* is highly susceptible to *Aeromonas hydrophila* infection after suppression of proPO [14]. The absence of proPO in the kuruma shrimp, *Marsupenaeus japonicus*, significantly increases mortality and bacterial load in the hemolymph [15]. In the shrimp *Penaeus monodon*, co-silencing of *Pm*proPO1 and 2 also had increased mortality after the bacterial *V*. *harveyi* and the fungus *Fusarium solani* infections [16,17]. In addition, the melanization reaction products from shrimp hemocyte also had *in vitro* antimicrobial activities against Gram-negative bacteria (*V*. *harveyi* and *V*. *parahaemolyticus*), Gram-positive bacteria (*Bacillus subtilis*) and fungus (*F*. *solani*) [17].

Because the highly toxic intermediates generated from the melanization process are harmful to pathogens as well as the host tissue itself, this process must be tightly regulated and localized [18]. Thus, several serine proteinases (SPs) and serine proteinase homologs (SPHs) are involved in controlling the proteolysis steps. SPHs are SP-like enzymes that contain one or more clip-domain(s) at the N-terminus and an SP-like domain at the C-terminus, but the catalytic triad serine has been substituted with glycine, and therefore, SPHs lack proteolysis activity. Several SPHs have been identified in insects and crustaceans and their roles in the proPO cascade have been characterized. In insects, the *Anopheles gambiae* SPH, CLIPA8, is required for melanization because knockdown of CLIPA8 will completely abolish melanin synthesis on the surface of the fungus *Beauveria bassiana* [12]. The *Manduca sexta* SPHs (SPH1 and SPH2) are also required for generating the active PO and enhancing PO activity via a collaborative function with the proPO activating proteinase-1 (PAP-1) and PAP-3 [19,20]. Moreover, a non-clip domain-containing SPH-3 of *M*. *sexta* is required for PO activation, nodule formation, and gene transcription of antimicrobial effectors [21]. The proPO-activating factor-II (PPAF-II) of *Holotrichia diomphalia* has been characterized as the co-activator of PPAF-I, a clip-domain SP that cleaves proPO into PO, and the clip domain of PPAF-II also plays a role in proPO activation by interacting with PO through its central cleft [22,23]. In *Tenebrio molitor*, SPH1 but not SPH2 is a proPO activating cofactor that functions together with active PO to regulate melanin production by forming an active melanization complex that efficiently produces melanin on the surface of bacteria and has strong antibacterial activity [24,25]. In addition to proPO activation, SPHs also possess other functions related to embryonic development such as in *Drosophila* [26] and are also involved in PRP recognition. The crayfish *P*. *leniusculus* masquerade-like protein *Pl*-MasI functions as a PRP that binds LPS, β-1,3-glucan, Gram-negative bacteria and yeast and is involved in the clearance of microorganisms [27], whilst *Pl*SPH1 and *Pl*SPH2 may act as the PRP to the Gram-positive bacteria cell wall component PGN [28]. In the mud crab *Scylla paramamosain*, *Sp-*SPH can also bind various microbial cell wall components such as PGN, LPS and β-1,3-glucans [29].

Our research group has identified and characterized several important proteins in the shrimp proPO system [16,30–33] including the *P*. *monodon* masquerade-like SPH called *Pm*MasSPH1 that acts as a multifunctional immune protein [34,35]. Transcription of *Pm*MasSPH1 has been shown to be induced in response to *V*. *harveyi* infection [34]. Furthermore, the C-terminal SP-like domain of *Pm*MasSPH1 can act as a cell adhesive molecule and binds to *V*. *harveyi*, LPS and virus [35,36]. The N-terminal region of *Pm*MasSPH1, containing clip and glycine-rich domains, also has antimicrobial activity against Gram-positive bacteria [35]. In the present study, a newly identified *Pm*MasSPH2 is described and both *Pm*MasSPH1 and *Pm*MasSPH2 are further investigated for their roles in activation of the proPO system using RNA interference (RNAi) and protein-protein interaction approach. Moreover, the PRP properties for *Pm*MasSPHs in recognizing and binding to the Gram-positive bacterial cell wall component PGN are also examined.

## Materials and Methods

### Sample preparation

Specific pathogen-free (SPF) *Penaeus monodon* (~4 g, fresh weight) were purchased from the Shrimp Genetic Improvement Center, BIOTEC, Thailand. Shrimp were reared in an aerated tank with seawater at 20 ppt salinity for 7 days before the experiments began. The hemolymph was collected and total RNA was extracted using the TRI Reagent (Molecular Research Center) following the manufacturer’s protocol. First-strand cDNA was synthesized using the ImProm-II Reverse Transcriptase System kit (Promega) with 1.5 μg of total RNA and 0.5 μg of oligo(dT)_15_ primer. For the gene expression analysis at different developmental stages, RNA extraction and cDNA synthesis as described above was done on three individual shrimp from four stages including nauplius 3 (N3), protozoea 2 (Z2), mysis 2 (M2) and post-larvae 15 (PL15).

### Sequence analysis

The sequence similarity search was performed using the BLAST algorithm (http://www.ncbi.nlm.nih.gov/BLAST/). The putative signal peptide cleavage site and the structural protein domains were predicted using the SignalP 3.0 server (http://www.cbs.dtu.dk/services/SignalP/) and the simple modular architecture research tool (SMART) (http://smart.embl-heidelberg.de/), respectively. Multiple amino acid sequence alignments were carried out using the ClustalW2 program (http://www.ebi.ac.uk/Tools/clustalw2/). The phylogenetic tree was created in Molecular Evolutionary Genetics Analysis (MEGA) version 5.2.

### Double-stranded RNAs (dsRNAs) preparation and gene silencing

dsRNA was generated and purified as described previously [16] using gene specific primers (*Pm*SPH1-T7F, *Pm*SPH1-T7R, *Pm*SPH1-F and *Pm*SPH1-R for *Pm*MasSPH1, *Pm*SPH2-T7F, *Pm*SPH2-T7R, *Pm*SPH2-F and *Pm*SPH2-R for *Pm*MasSPH2, and GFPT7-F, GFPT7-R, GFP-F and GFP-R for GFP) (Table 1). For the gene silencing experiment, shrimp (approximately 4 g) were intramuscularly injected with *Pm*MasSPH1 or *Pm*MasSPH2 dsRNA at a concentration of 2 μg/g shrimp. Shrimp injected with GFP dsRNA and 150 mM NaCl solution served as the non-related dsRNA and negative controls, respectively. 24 h after injection, shrimp were injected again with corresponding dsRNA and NaCl containing 20 μg of lipopolysaccharide (LPS) (Sigma-Aldrich) and 20 μg of laminarin (β-1,3-glucan; Sigma-Aldrich). Shrimp were reared for 48 h after second injection prior to further analysis.

**Table 1:** Primers used in the experiments.

Primer name	Sequence (5’-3’)	Purpose
*Pm*SPH1-T7F	GGATCCTAATACGACTCACTATAGGTTATAACGGACGGCGCA	RNAi
*Pm*SPH1-T7R	GGATCCTAATACGACTCACTATAGGCCAGCAGGTGTCGTAGT	RNAi
*Pm*SPH1-F	TTATAACGGACGGCGCAGGC	RNAi
*Pm*SPH1-R	CCAGCAGGTGTCGTAGTCGAAT	RNAi
*Pm*SPH2-T7F	GGATCCTAATACGACTCACTATAGGGTGTGCATTAGCTGTGGCCGTC	RNAi
*Pm*SPH2-T7R	GGATCCTAATACGACTCACTATAGGAGCCCGAGCGCTGAATGGGTAC	RNAi
*Pm*SPH2-F	GTGTGCATTAGCTGTGGCCGTC	RNAi
*Pm*SPH2-R	AGCCCGAGCGCTGAATGGGTAC	RNAi
GFPT7-F	TAATACGACTCACTATAGGATGGTGAGCAAGGGCGAGGA	RNAi
GFPT7-R	TAATACGACTCACTATAGGTTACTTGTACAGCTCGTCCA	RNAi
GFP-F	ATGGTGAGCAAGGGCGAGGA	RNAi
GFP-R	TTACTTGTACAGCTCGTCCA	RNAi
*Pm*SPH1rtF	TACCCTCACCAGGACAGGAACG	RT-PCR
*Pm*SPH1rtR	CTGGAAGAAAGATCCGAGCCGA	RT-PCR
*Pm*SPH2-322-F	CAGGCGGAGAGTGTGGCATC	RT-PCR
*Pm*SPH2-322-R	TTGTGCTCGCCCAGACGGAC	RT-PCR
*Pm*proPO1-F	GGTCTTCCCCTCCCGCTTCG	RT-PCR
*Pm*proPO1-R	GCCGCAGGTCCTTTGGCAGC	RT-PCR
*Pm*proPO2-F	GCCAAGGGGAACGGGTGATG	RT-PCR
*Pm*proPO2-R	TCCCTCATGGCGGTCGAGGT	RT-PCR
PPAE1-F	CGTCTGCTTCATTGAGGGAGTG	RT-PCR
PPAE1-R	GTAGTAGATGGTGCCCCAGCCT	RT-PCR
PPAE2-F	GCGGCGGTCACGCTCCTTGTTC	RT-PCR
PPAE2-R	ACTCTCGGGGGCACGCTTGTTG	RT-PCR
PEN3-F	GGTCTTCCTGGCCTCCTTCG	RT-PCR
PEN3-R	TTTGCATCACAACAACGTCCTA	RT-PCR
PEN5-F	TTGGTCTATGCTTTGCAAGG	RT-PCR
PEN5-R	ACAGATAGTTAAAGTGAAAGAC	RT-PCR
crustin*Pm*1-F	CTGCTGCGAGTCAAGGTATG	RT-PCR
crustin*Pm*1-R	AGGTACTGGCTGCTCTACTG	RT-PCR
Crus-like*Pm*-F	CGGCAGGTGTCCACAGATTCG	RT-PCR
Crus-like*Pm*-R1	AATTGATGAGTCGAACATGCAGGCCTAT	RT-PCR
EF1α-F	GGTGCTGGACAAGCTGAAGGC	RT-PCR
EF1α-R	CGTTCCGGTGATCATGTTCTTGATG	RT-PCR
*Pm*MasSPH2NcoI-F	CATGCCATGGGCCAGAACAACCAGAACGTAAGGCT	Recombinant protein expression
*Pm*MasSPH2XhoI-R	CGCCTCGAGCTAATGATGATGATGATGATGAAATCTCACGAATTGCCTAATAAAG	Recombinant protein expression
*Pm*PPAE2NcoI-F	CATGCCATGGGCCATCATCATCATCATCATAAAATATTCGGTGGCGAAGCTAC	Recombinant protein expression
*Pm*PPAE2NotI-R	ATAAGAATGCGGCCGCCTAAGGTTTGAGATTCTGCACG	Recombinant protein expression
*Pm*SPH1-Y2H-F	GGAATTCCATATGATGCGGGTGTTGGCAGT	Yeast two-hybrid
*Pm*SPH1-Y2H-R	CCGCTCGAGAATAAATCTTCCGTAGTCCCA	Yeast two-hybrid
PPAE2-Y2H-F	CATGCCATGGGCCATCATCATCATCATCATACGAGAGATCGAAGGCAAGCCA	Yeast two-hybrid
PPAE2-Y2H-R	CGCCTCGAGCTAAGGTTTGAGATTCTGCACG	Yeast two-hybrid

### Hemolymph phenoloxidase (PO) activity assay

The total PO activity in shrimp hemolymph was measured as described previously [16]. 48 h after the second dsRNA injection, hemolymph was collected without using any anticoagulant. Total protein concentration was quantified using the Bradford protein assay kit (Bio-Rad). Subsequently, 2 mg total protein was analyzed for PO activity using L-3,4-dihydroxyphenylalanine (L-DOPA) as the substrate. PO activity was defined as ΔA_490_ per mg total protein/min. The results were analyzed from independent three experiments using a one-way analysis of variance (ANOVA).

### Determining bacterial load in *Pm*MasSPHs silenced shrimp

To investigate the involvement of *Pm*MasSPHs in bacterial clearance, shrimp were injected with dsRNA specific to *Pm*MasSPH1, *Pm*MasSPH2 and control GFP as described above. For the second injection, shrimp were given *V*. *harveyi* 639 at a concentration of 2×10^5^ colony forming units (CFUs). 6 h after bacteria challenge, the hemolymph was collected from each shrimp and serially diluted on LB agar plates and incubated at 30°C overnight. The number of colonies formed on the plate was calculated as CFU/ml.

### Gene expression analysis in *Pm*MasSPHs silenced shrimp

To test whether gene silencing of *Pm*MasSPHs could affect the expression of other immune-related gene transcripts, gene expression analysis was carried out in *Pm*MasSPHs silenced shrimp. The hemolymph was collected 48 h after dsRNA injection and then RNA was extracted and cDNA was made as described previously. Gene expression levels of several proPO-related proteins (*Pm*proPO1, *Pm*proPO2, *Pm*PPAE1 and *Pm*PPAE2) and antimicrobial proteins (*Penmon*PEN3, *Penmon*PEN5, crustin*Pm*1 and Crus-like*Pm*) were determined by semi-quantitative RT-PCR using the gene specific primers found in Table 1. The PCR conditions were as follows: 94°C 1 min, followed by 25 cycle of 94°C for 30 s, 55°C for 30 s and 72°C for 1 min, and finally 72°C for 5 min for the final step. PCR products were visualized by agarose gel electrophoresis and band intensity was analyzed using a Gel-Pro Analyzer. The expression levels of each gene are shown as relative expression normalized to EF1-α. The statistical significance of the triplicate experiments was determined by one-way ANOVA.

### Expression and purification of recombinant proteins

The C-terminal SP-like domain of *Pm*MasSPH2 and the SP domain of *Pm*PPAE2 were amplified using *Pfu* DNA polymerase (Promega) with the primer pairs *Pm*MasSPH2NcoI-F / *Pm*MasSPH2XhoI-R and *Pm*PPAE2NcoI-F / *Pm*PPAE2NotI-R, respectively (Table 1). The PCR products were purified and cloned into the expression vector pET28b (Novagen). The His-tagged recombinant plasmids of the C-terminal SP-like domain of *Pm*MasSPH1 [35], *Pm*MasSPH2 (this study), the C-terminal SP domain of *Pm*PPAE1 [30] and *Pm*PPAE2 (this study) were used to express recombinant proteins in *Escherichia coli* Rosetta (DE3) pLysS. After induction with 1 mM IPTG at 37°C for 6 h, bacterial cells were harvested, re-suspended in 20 mM Tris-HCl (pH 8.0), then disrupted by sonication. The supernatant containing soluble protein was purified by nickel affinity chromatography (Ni-NTA Agarose; QIAGEN). In case inclusion bodies were obtained, protein was purified under denaturing conditions with 8 M urea in a Ni-NTA affinity column. The purified protein was subsequently refolded by dialysis with 20 mM Tris-HCl (pH 8.0). The protein concentration was then determined using a Bradford protein assay kit (Bio-Rad).

### Quantitative determination of *Pm*MasSPHs binding activity to PGN

An ELISA assay was performed to investigate the binding activities of both *Pm*MasSPH1 and *Pm*MasSPH2 to the bacterial cell wall component PGN. A 96-well plate was coated with 2 μg PGN from *Bacillus subtilis* (InvivoGen) per well in 50 μl water and then incubated at 37°C overnight. After fixing at 60°C for 2 h and blocking with 5% BSA in TBS for 1 h, the PGN-coated plate was washed with TBS three times. 100 μl of recombinant protein at various concentrations was added in each well and incubated at 4°C overnight. After washing, 100 μl of a mouse anti-His antibody (1:5000) was added in each well and incubated at room temperature for 3 h. After washing, the alkaline phosphatase-conjugated goat anti-mouse antibody (1:10000) was added and incubated for 1 h. The plate was then washed twice with TBST and twice with water before adding the substrate (AP Substrate Kit; Bio-Rad). The results were analyzed by Scatchard plot analysis. The dissociation constant (*K*_d_) and the maximum binding (A_max_) was calculated from the Y-intercept and slope of linear equation as 1/A = *K*_d_/A_max_[L]+1/A_max_ (A is defined as the absorbance at 405 nm and [L] is the protein concentration).

### Protein-protein interaction assay

To determine if there is an interaction between *Pm*MasSPH1 and *Pm*PPAE2, we performed a yeast two-hybrid analysis from Matchmaker GAL4 Two-Hybrid System (Clontech). Full-length cDNA encoding *Pm*MasSPH1 was cloned and fused in-frame with the activation domain (AD) of the pGADT7 vector by PCR amplification using *Pm*SPH1-Y2H-F and *Pm*SPH1-Y2H-R primers (Table 1). Full-length cDNA encoding *Pm*PPAE2 was fused in-frame with the GAL4 DNA binding domain (BD) of the pGBKT7 vector using PPAE2-Y2H-F and PPAE2-Y2H-R primers (Table 1). The recombinant plasmids of *Pm*MasSPH1/AD and *Pm*PPAE2/BD were co-transformed into the *Saccharomyces cerevisiae* strain AH109 using the lithium acetate/dimethyl sulfoxide method [37]. Transformed yeast cells were selected on minimal media plates lacking leucine and tryptophan (-L/-W). Positive interactions were indicated by growth on high stringency medium lacking adenine, histidine, leucine and tryptophan (-A/-H/-L/-W) and by a blue color change from X-α-gal (Apollo Scientific) present in the media. The empty vector pGADT7 was also co-transformation as negative control and an interaction between murine p53 bait fusion (pVA3) and SV40 prey fusion (pTD1) served as a positive control (Clontech).

In addition to the yeast two-hybrid assay, co-immunoprecipitation (co-IP) was also performed between the r*Pm*MasSPHs and r*Pm*PPAEs that were produced in *E*. *coli* as described above. The procedure was conducted according to Isono and Schwechheimer [38] with a slight modification. Briefly, to test if r*Pm*MasSPH1 and r*Pm*PPAE2 interact, 40 μg of purified anti-SP-like domain of *Pm*MasSPH1 antibody was mixed with 40 μl of protein A agarose (Sigma) in 20 mM Tris-HCl (pH 8.0) and incubated at 4°C for 3 h. 50 μM of r*Pm*MasSPH1 and 50 μM of the *Pm*PPAE2 were incubated together at 4°C for 3 h and then loaded onto the antibody-tagged resin and further incubated at 4°C for 3 h. After washing 3 times with freshly prepared wash buffer (50 mM Tris-HCl (pH 7.5), 100 mM NaCl, 10% (v/v) glycerol and 0.05% (v/v) Triton X-100), the protein complex was eluted with 2X SDS-PAGE sample loading buffer. The protein complex was then separated by SDS-PAGE, transferred to a nitrocellulose membrane and detected with anti-His antibody. The co-IP assay between *Pm*MasSPH2 and *Pm*PPAEs was also performed using the purified anti-SP-like domain of *Pm*MasSPH2 antibody as described for *Pm*MasSPH1.

### Effect of *Pm*MasSPH1 and *Pm*PPAE2 on the PGN-triggered hemolymph PO activity *in vitro*

To identify whether *Pm*MasSPH1 and *Pm*PPAE2 are necessary for activation of proPO after infection by Gram-positive bacteria, PO activity was measured in the presence of PGN and the recombinant proteins. Fresh shrimp hemolymph (HL) was collected and the total protein concentration measured by the Bradford assay (Bio-Rad). Total reaction included 0.8 mg HL with the addition of 0.25 μg PGN and 1 μM of each recombinant protein (*Pm*PPAE2 and *Pm*MasSPH1 or BSA as the protein control). The CAC buffer (10 mM CAC containing 10 mM CaCl_2_) was added to a final volume of 174 μl. 26 μl of 3 mg/ml L-DOPA was added and the ΔA_490_ measured at 5 min. Each experimental treatment was performed in triplicate. Data were analyzed using a one-way analysis of variance (ANOVA) followed by Duncan’s test and a *p* < 0.05 was considered statistically significance.

## Results

### Sequence analysis of *Pm*MasSPH1 and *Pm*MasSPH2

**Fig 1:**
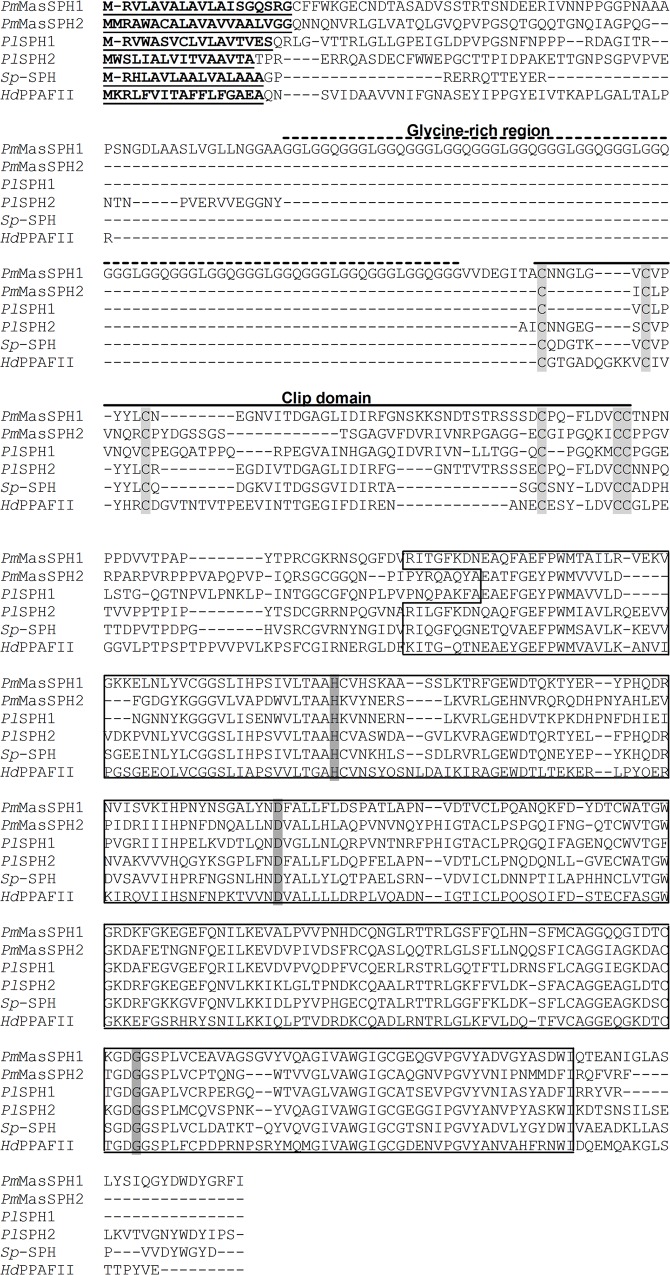
A multiple amino acid sequence alignment of *Pm*MasSPH1 and *Pm*MasSPH2 with other arthropod SPHs. The amino acid sequence of *Penaeus monodon Pm*MasSPH1 (ABE03741), *Pm*MasSPH2 (ACP19560), *Pacifastacus leniusculus Pl*SPH1 (AAX55746), *Pl*SPH2 (ACB41379), *Scylla paramamosain Sp*-SPH (ADG83846) and *Holotrichia diomphalia Hd*PPAFII (CAC12665) were collectively compared. The predicted signal peptides are in bold and underlined. The dash line indicates the glycine-rich domain of *Pm*MasSPH1. The conserved clip-domains are indicated below the line. The light-grey highlight indicates the cysteine residues in the clip-domains. The black box shows the conserved serine proteinase-like domain with the grey highlight indicating the catalytic triad (His, Asp, and Gly residues) in the domain.

**Fig 2:**
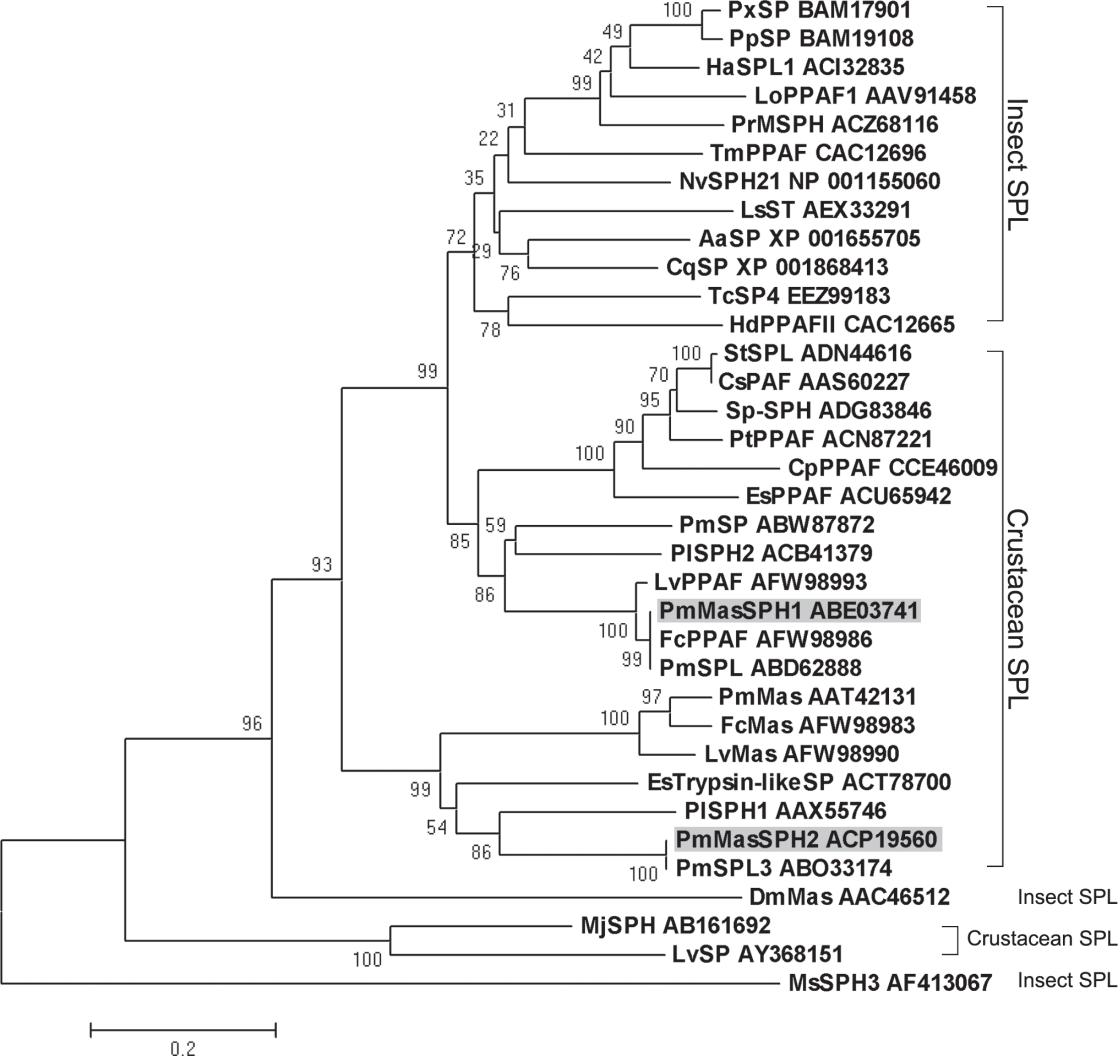
The phylogenetic relationship between serine proteinase domains from *Pm*MasSPH1 and *Pm*MasSPH2 and other serine proteinases. The deduced amino acid sequences of SP-domains from various clip-SPH species were used to generate a phylogenetic tree by the neighbor-joining method. Percent bootstrap values (1000 replicates) are shown at each branch point. *Pm*MasSPH1 (ABE03741); *Pm*MasSPH2 (ACP19560); *Pm*SP, *P*. *monodon* serine proteinase (ABW87872); *Pm*SPL, *P*. *monodon* serine proteinase-like protein (ABD62888); *Pm*SPL3 (ABO33174); *Pm*Mas, *P*. *monodon* mas-like protein (AAT42131); *Fc*PPAF, *Fenneropenaeus chinensis* prophenoloxidase activating factor (AFW98986); *Fc*Mas (AFW98983); *Lv*PPAF, *Litopenaeus vannamei* PPAF (AFW98993); *Lv*SP (AY368151); *Lv*Mas (AFW98990); *Pl*SPH1, *P*. *leniusculus* SPH1 (AAX55746); *Pl*SPH2 (ACB41379); *Ms*SPH3, *Manduca sexta* SPH3 (AF413067); *Hd*PPAFII, *Holotrichia diomphalia* PPAFII (CAC12665); *Mj*SPH, *Marsupenaeus japonicus* SPH (AB161692); *Nv*SPH21, *Nasonia vitripennis* SPH21 (NP_001155060); *Cq*SP, *Culexquin quefasciatus* SP (XP_001868413); *Aa*SP, *Aedes aegypti* SP (XP_001655705); *Pt*PPAF, *Portunus trituberculatus* PPAF (ACN87221); *Cs*PAF, *Callinectes sapidus* PAF (AAS60227); *Es*PPAF, *Eriocheir sinensis* PPAF (ACU65942); *Es*Trypsin-likeSP (ACT78700); *Ls*ST, *Lucilia sericata* salivary trypsin (AEX33291); *Cp*PPAF, *Cancer pagurus* PPAF (CCE46009); *Pp*SP, *Papilio polytes* SP(BAM19108); *Ha*SPL1, *Helicoverpa armigera* SPL1 (ACI32835); *Tm*PPAF, *Tenebrio molitor* PPAF (CAC12696); *St*SPL, *Scylla tranquebarica* SPL (ADN44616); *Lo*PPAF1, *Lonomia obliqua* PPAF1 (AAV91458); *Px*SP, *Papilio xuthus* SP (BAM17901); *Pr*MSPH, *Pieris rapae* MSPH (ACZ68116); *Tc*SP4, *Tribolium castaneum* SP4 (EEZ99183); *Dm*Mas, *Drosophila melanogaster* Mas (AAC46512); *Sp*-SPH, *S*. *paramamosain* SPH (ADG83846).

Based on the shrimp *P*. *monodon* EST database (http://pmonodon.biotec.or.th/) [39], three full-length cDNAs of clip SPHs (*Pm*MasSPH1, *Pm*MasSPH2 and *Pm*MasSPH3) have been isolated [6]. Among these, a masquerade-like SPH1 (*Pm*MasSPH1) of shrimp *P*. *monodon* has been cloned and its role in shrimp immunity has been characterized [34,35]. In this study, *Pm*MasSPH1 and the novel SPH *Pm*MasSPH2, were investigated to further elucidate their function in proPO activation. A full-length sequence of *Pm*MasSPH2 was obtained with an open reading frame of 1164 bp encoding 387 amino acid residues. The *Pm*MasSPH2 cDNA sequence was deposited in the GenBank database under accession number FJ620686. Apart from a signal peptide of 20 amino acids at the N-terminus, the mature protein (367 amino acids) has the calculated molecular mass of 39.37 kDa and the predicted p*I* of 7.52. The conserved clip-domain and serine proteinase (SP)-like domain with the catalytic triad (His_184_, Asp_234_ and Gly_338_) were also found in *Pm*MasSPH2, *Pm*MasSPH1 and other SPHs (Fig. 1). BLAST analysis of *Pm*MasSPH1 and *Pm*MasSPH2 showed high homology to the SPs of crustaceans and insects. High similarity and conserved function involved in proPO cascade have also been reported for the crayfish *Pacifastacus leniusculus Pl*SPH1 and *Pl*SPH2, crab *Scylla paramamosain Sp*-SPH and beetle *Holotrichia diomphalia Hd*PPAF-II [28,29,40], and thus, we aligned these amino acid sequences with both *Pm*MasSPH1 and *Pm*MasSPH2 (Fig. 1). Interestingly, *Pm*MasSPH1 and *Pm*MasSPH2 have only 34.6% sequence similarity but high homology was observed across other species. For instance, *Pm*MasSPH1 has 55.5% similarity to *Pl*SPH2, and *Pm*MasSPH2 and *Pl*SPH1 share 68.2% similarity. Sequence comparison of only the SP-like domain showed consistency in similarity among selected species. The SP-like domains of *Pm*MasSPH1 and *Pm*MasSPH2, *Pm*MasSPH1 and *Pl*SPH2, and *Pm*MasSPH2 and *Pl*SPH1 share 51.6%, 73.6% and 78.9% similarity, respectively. A phylogenetic analysis of the SP-like domain of insect and crustacean PPAFs was also carried out to examine the evolutionary relationships. A phylogenetic tree constructed using the Neighbor-Joining method revealed that *Pm*MasSPH1 and *Pm*MasSPH2 were grouped in a clade of crustacean PPAFs (Fig. 2). *Pm*MasSPH1 is closely related to *P*. *monodon* serine proteinase-like protein (*Pm*SPL) and other shrimp PPAFs and is grouped in the same clade as *Pl*SPH2. In contrast, *Pm*MasSPH2 is more closely linked to *Pm*SPL3 and *Pl*SPH1 (Fig. 2).

### Gene expression in various developmental stages of shrimp larva

**Fig 3:**
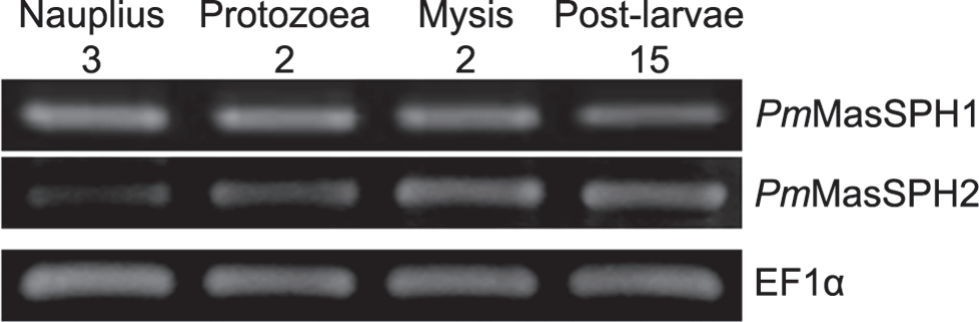
Expression of *Pm*MasSPH1 and *Pm*MasSPH2 transcripts at various stages of shrimp larval development. Expression profiles of *Pm*MasSPH1 and *Pm*MasSPH2 were examined at four larval stages including nauplius 3 (N3), protozoea 2 (Z2), mysis 2 (M2) and post-larvae 15 (PL15) by semi-quantitative RT-PCR. The elongation factor 1-α (EF1-α) served as an internal control. Each lane represents the result of individual shrimp (n = 3).

We next examined the expression profile of *Pm*MasSPH1 and *Pm*MasSPH2 transcripts to better understand regulation of immune genes during shrimp development. Gene expression patterns of *Pm*MasSPH1 and *Pm*MasSPH2 were obtained and compared to the internal EF1-α control by RT-PCR analysis at four shrimp larval developmental stages including nauplius 3 (N3), protozoea 2 (Z2), mysis 2 (M2) and post-larvae 15 (PL15). We found that both *Pm*MasSPH1 and *Pm*MasSPH2 were expressed at all the developmental stages we tested but with different expression patterns. Expression levels of the *Pm*MasSPH1 transcripts were stable at all stages, while the *Pm*MasSPH2 transcripts were lower at the early stages and then gradually increased as development progressed (Fig. 3).

### Knockdown of *Pm*MasSPHs decreases hemolymph PO activity

**Fig 4:**
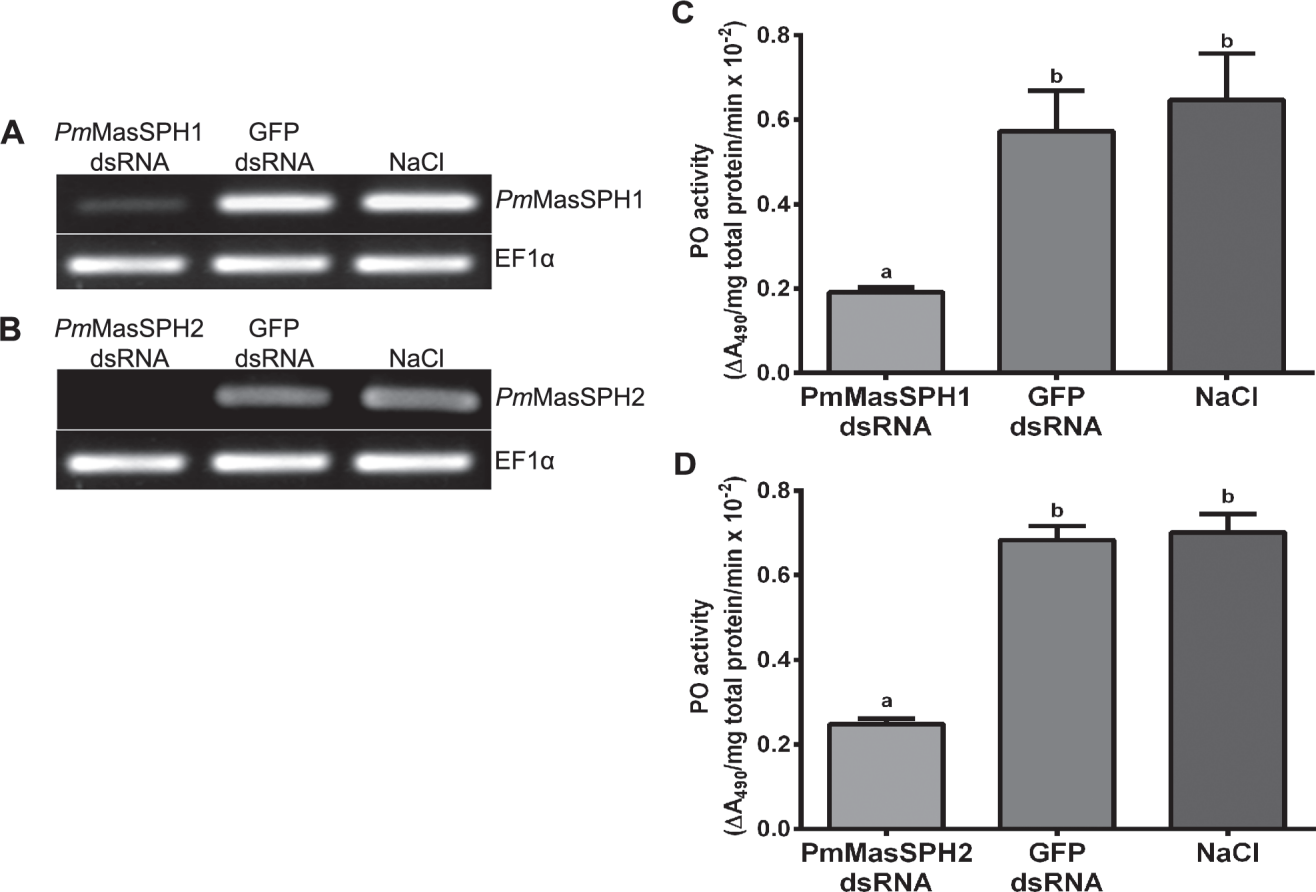
Hemolymph PO activity of *Pm*MasSPH1 and *Pm*MasSPH2 silenced shrimp. The efficiency of gene silencing of *Pm*MasSPH1 and *Pm*MasSPH2 was determined by semi-quantitative RT-PCR. Transcription levels of *Pm*MasSPH1 (A) and *Pm*MasSPH2 (B) were examined in shrimp injected with the corresponding dsRNA. Shrimp injected with GFP dsRNA or NaCl served as control groups. Expression of EF1-α was used as an internal control. Each band represents the pooled cDNA of triplicate samples of each treatment group. Hemolymph PO activity of the *Pm*MasSPH1 (C) and *Pm*MasSPH2 (D) silenced shrimp was also examined. Shrimp injected with GFP dsRNA or NaCl served as control groups. The total hemolymph PO activity was measured as ΔA_490_/mg total protein/min. The data are shown as the mean ± standard deviation (error bars) from three independent experiments. Significantly different means (*p* < 0.05) are indicated by the lower case letters (a, b) above each bar.

Because several proteins together participate in the proPO cascade to defend against invading pathogens, we evaluated the involvement of *Pm*MasSPH1 and *Pm*MasSPH2 in proPO activation by RNAi-mediated gene silencing. Shrimp were injected twice with dsRNA against *Pm*MasSPH1 or *Pm*MasSPH2 with GFP dsRNA or NaCl solution used as control groups. 48 h after the second injection, shrimp hemolymph was collected and gene transcription analyzed. Our results show that shrimp receiving *Pm*MasSPH1 or *Pm*MasSPH2 dsRNA efficiently suppressed *Pm*MasSPH1 or *Pm*MasSPH2 transcript expression (Fig. 4A and B). Injection of GFP dsRNA or NaCl solution had no effect on *Pm*MasSPH1 or *Pm*MasSPH2 mRNA transcript levels.

To examine whether *Pm*MasSPH1 and *Pm*MasSPH2 are involved in the activation of proPO, the total hemolymph PO activity of *Pm*MasSPH1 or *Pm*MasSPH2 silenced shrimp was measured using L-DOPA as a substrate. The hemolymph PO activity of *Pm*MasSPH1 and *Pm*MasSPH2 knockdown shrimp decreased significantly by 66.5% and 63.7%, respectively. No significant change in PO activity was observed in the control groups (*p* < 0.05) (Fig. 4C and D). These results indicate that *Pm*MasSPH1 and *Pm*MasSPH2 are involved in proPO activation in shrimp.

### *Pm*MasSPH silencing increase bacterial load in shrimp hemolymph

**Fig 5:**
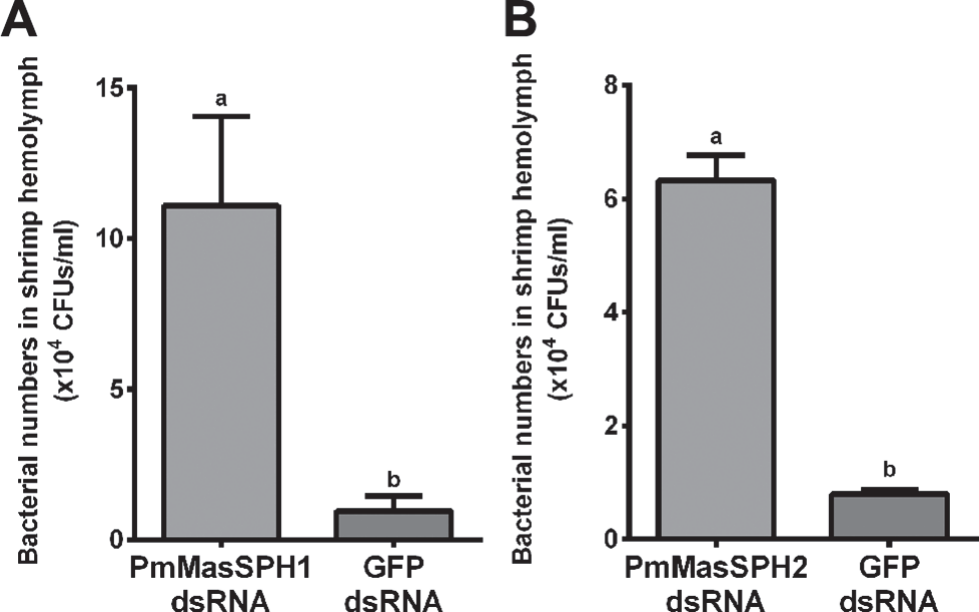
Increase in bacterial load in hemolymph from *Pm*MasSPHs silenced shrimp. Shrimp were injected twice with dsRNA specific to *Pm*MasSPH1 (A) or *Pm*MasSPH2 (B). At the second dsRNA injection, shrimp were also injected with *V*. *harveyi* (2×10^5^ CFU/shrimp). Control groups given injections of GFP dsRNA or NaCl. The number of viable bacteria in the knockdown shrimp hemolymph is shown as the bacterial CFUs 6 h after challenge. The data are shown as the mean ± standard deviation derived from three independent experiments. Means with lower case letters above each bar indicate significant differences (*p* < 0.05).

The above results clearly demonstrate that both *Pm*MasSPH1 and *Pm*MasSPH2 have potential roles in proPO activation. Therefore, we investigated whether *Pm*MasSPH1 and *Pm*MasSPH2 have immune activity against shrimp pathogenic bacteria. The *Pm*MasSPH1 or *Pm*MasSPH2 silenced shrimp were challenged with pathogenic *V*. *harveyi* at 2 × 10^5^ CFU/shrimp. As a control, shrimp injected with GFP dsRNA were also challenged with *V*. *harveyi*. 6 h after bacterial challenge, hemolymph from individual shrimp was collected and the CFU of *V*. *harveyi* was determined. Our results show that the number of viable bacterial CFUs in the *Pm*MasSPH1 and *Pm*MasSPH2 silenced shrimp increased 11.5 and 7.9-fold, respectively, compared to the control shrimp injected with GFP dsRNA (Fig. 5A and B). It is worth noting that the *Pm*MasSPH1 silenced shrimp have a higher bacterial hemolymph load than the *Pm*MasSPH2 silenced shrimp. These results suggest that both *Pm*MasSPH1 and *Pm*MasSPH2 are involved in the process of hemolymph bacterial clearance in the shrimp *P*. *monodon*.

### *Pm*MasSPH1 silencing alters expression of other immune genes

**Fig 6:**
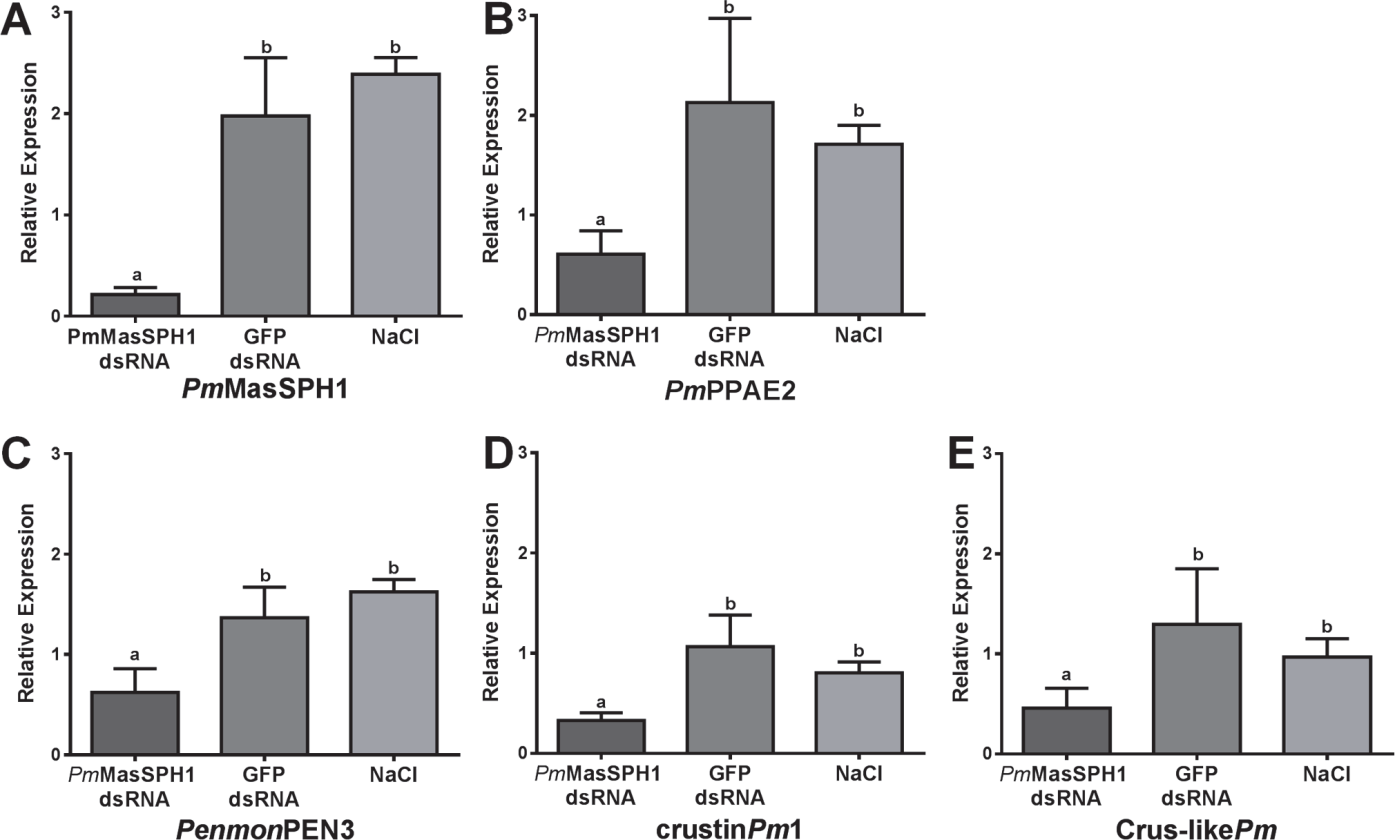
The effect of *Pm*MasSPH1 gene silencing on expression of other immune genes. Transcription levels of *Pm*MasSPH1 (A), *Pm*PPAE2 (B), *Penmon*PEN3 (C), crustin*Pm*1 (D), and Crus-like*Pm* (E) were analyzed in *Pm*MasSPH1 silenced shrimp and control groups (GFP dsRNA or NaCl treatment). Each transcript is expressed as the relative expression normalized to the EF1-α transcript. Each bar is displayed as the mean ± standard deviation (error bar). The lower case letters represent significant differences between treatments.

Several studies in insect immunity have shown evidence that a single gene does not strictly regulate one pathway but can play a role in several pathways. Previous studies in the lepidopteran *Manduca sexta* showed that SPH-3 is not only required for proPO activation but is also important for transcription of other immune effector genes [21]. We therefore, examined the effect of *Pm*MasSPHs silencing on the expression of other immune genes by semi-quantitative RT-PCR. The hemocyte cDNAs from *Pm*MasSPH1 or *Pm*MasSPH2 silenced shrimp was examined for transcript expression of other proPO-related genes (*Pm*proPO1, *Pm*proPO2, *Pm*PPAE1 and *Pm*PPAE2) and antimicrobial peptide genes (*Penmon*PEN3, *Penmon*PEN5, Crustin*Pm*1 and Crus-like*Pm*), with EF1-α as an internal control. Additionally, expression of these other immune-related genes was examined in the control groups injected with GFP dsRNA and NaCl. As shown in Fig. 6, shrimp injected with *Pm*MasSPH1 dsRNA have decreased expression of *Pm*MasSPH1 (89%), *Pm*PPAE2 (71%) (Fig. 6B), *Penmon*PEN3 (54%) (Fig. 6C), Crustin*Pm*1 (69%) (Fig. 6D) and Crus-like*Pm* (65%) (Fig. 6E) when compared to GFP dsRNA control shrimp. This suppression of immune genes by *Pm*MasSPH1 silencing was not caused by an off-target effect of RNAi because sequence comparison between the region corresponding to *Pm*MasSPH1 dsRNA and the other immune-related genes have no significant similarity (data not shown). *Pm*MasSPH2 gene silencing was also performed in the same manner as *Pm*MasSPH1. We found that only the *Pm*MasSPH2 transcripts (98%) were suppressed in this model, while expression of other immune-related genes did not change (data not shown). Taken together, *Pm*MasSPH1 appeared to be required for proPO activation and is involved in the synthesis of shrimp antimicrobial peptides.

### *In vitro* binding of *Pm*MasSPH1 and *Pm*MasSPH2 to PGN

**Fig 7:**
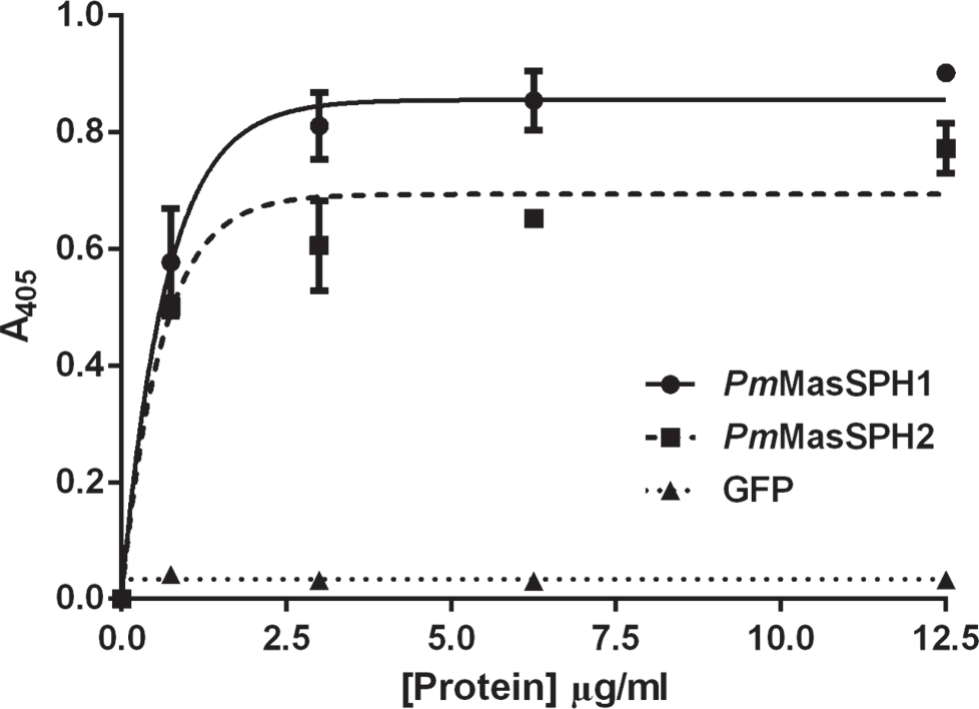
Ability of r*Pm*MasSPHs to bind a Gram-positive bacterial cell wall component. The binding ability of r*Pm*MasSPH1 or r*Pm*MasSPH2 (at varying concentrations 0–12.5 μg/ml) to *B*. *subtilis* PGN was quantified by ELISA assay. rGFP was included as a recombinant protein control. The data at each point was fitted to the trend line and is shown as the mean ± standard deviation from three individual experiments.

It has been shown that *Pm*MasSPH1 might act as a PRP because it can bind to the Gram-negative bacteria *V*. *harveyi*, bacterial cell wall component LPS, and virus [35,36]. However, it is unknown if *Pm*MasSPHs binds to Gram-positive bacteria. In this study, *Pm*MasSPH1 and *Pm*MasSPH2 were examined for a role in recognition of peptidoglycan (PGN), a Gram-positive bacterial cell wall component. An ELISA assay was used to quantitatively measure the binding activity between the recombinant proteins of mature *Pm*MasSPH1 or mature *Pm*MasSPH2 (with varying concentrations of 0–12.5 μg/ml) with 2 μg of ligand PGN from *Bacillus subtilis*. GFP served as a recombinant control protein. As shown in Fig. 7, the r*Pm*MasSPH1 and r*Pm*MasSPH2 directly bound PGN in a concentration-dependent manner. The dissociation constant (*K*_d_) of r*Pm*MasSPH1-PGN and r*Pm*MasSPH2-PGN were calculated as 6.51x10^-9^ M and 5.79x10^-9^ M, respectively. These results clearly indicate that *Pm*MasSPH1 and *Pm*MasSPH2 have a functional role as PRPs for PGN.

### *Pm*MasSPH1 interacts with *Pm*PPAE2

**Fig 8:**
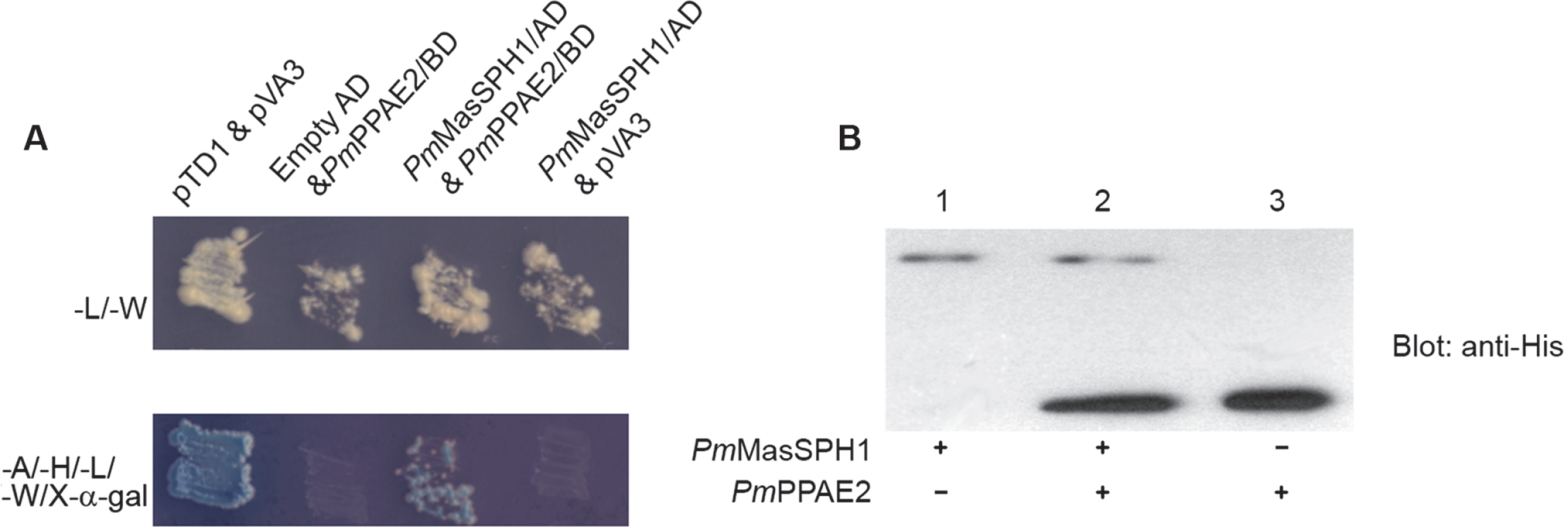
Interaction of *Pm*MasSPH1 with *Pm*PPAE2. The protein-protein interaction between *Pm*MasSPH1 and *Pm*PPAE2 were analyzed by yeast two-hybrid and co-IP assays. (A) The yeast two-hybrid results indicate the presence of BD and AD plasmids in transformed cells growing on an-L/-W plate. A putative interaction between *Pm*MasSPH1/AD and *Pm*PPAE2/BD as indicated by growth and blue color on the selective media-A/-H/-L/-W/X-α-gal. The positive control consisted of an interaction between murine p53 bait fusion (pVA3) and SV40 prey fusion (pTD1). The negative controls consisted of yeast cells containing *Pm*PPAE2/BD with an empty AD vector or pVA3 vector and *Pm*MasSPH1/AD. (B) The interaction between *Pm*MasSPH1 and *Pm*PPAE2 was confirmed by co-IP assay. The immunoprecipitated complex between r*Pm*MasSPH1 and r*Pm*PPAE2 was detected using the monoclonal anti-His antibody (lane 2). The r*Pm*MasSPH1 or r*Pm*PPAE2 alone (lanes 1 and 3, respectively) was used as a protein indicator.

Because it has been demonstrated that some insect SPHs function as co-factors of the terminal PPAEs of proPO activation [11], it is important to investigate whether *Pm*MasSPHs interact with *Pm*PPAEs. Because *Pm*PPAE2 changed expression when *Pm*MasSPH1 was silenced, we examined if there is a protein-protein interaction between *Pm*MasSPH1 and *Pm*PPAE2 using yeast two-hybrid and co-immunoprecipitation (co-IP) assays. In the yeast two-hybrid analysis, both *Pm*MasSPH1/AD and *Pm*PPAE2/BD plasmids were co-transformed into the yeast AH109 strain and plated on selective media. Our results show that *Pm*MasSPH1 specifically binds to *Pm*PPAE2 as indicated by blue colony formation on the selective media-A/-H/-L/-W/X-α-gal (Fig. 8A, lane *Pm*MasSPH1/AD and *Pm*PPAE2/BD). In addition, no self-activation occurred when *Pm*PPAE2/BD was co-transformed with an empty AD plasmid (Fig. 8A, lane empty AD and *Pm*PPAE2/BD). Co-transformation of *Pm*MasSPH1/AD with the control pVA3 plasmid was also performed to confirm that *Pm*MasSPH1 did not interact with the BD plasmid. The yeast two-hybrid assay suggests a genuine interaction between *Pm*MasSPH1 and *Pm*PPAE2.

To further confirm the protein-protein interaction, a co-IP assay with r*Pm*MasSPH1 and r*Pm*PPAE2 was carried out using anti-SPH1-conjugated protein A agarose beads to precipitate the protein complex. As shown in Fig. 8B lane 2, both *Pm*MasSPH1 and *Pm*PPAE2 were observed in the elute protein fraction indicating an interaction between the proteins. In addition, other pairwise protein interactions (r*Pm*MasSPH1 and r*Pm*PPAE1, r*Pm*MasSPH2 and r*Pm*PPAE1, and r*Pm*MasSPH2 and r*Pm*PPAE2) were also examined by co-IP assay. However, no other positive interactions were found (data not shown). Altogether, the yeast two-hybrid and co-IP analyses indicate a direct interaction between *Pm*MasSPH1 and *Pm*PPAE2.

### Enhancement of PGN-triggered hemolymph PO activity by *Pm*MasSPH1 and *Pm*PPAE2 *in vitro*

**Fig 9:**
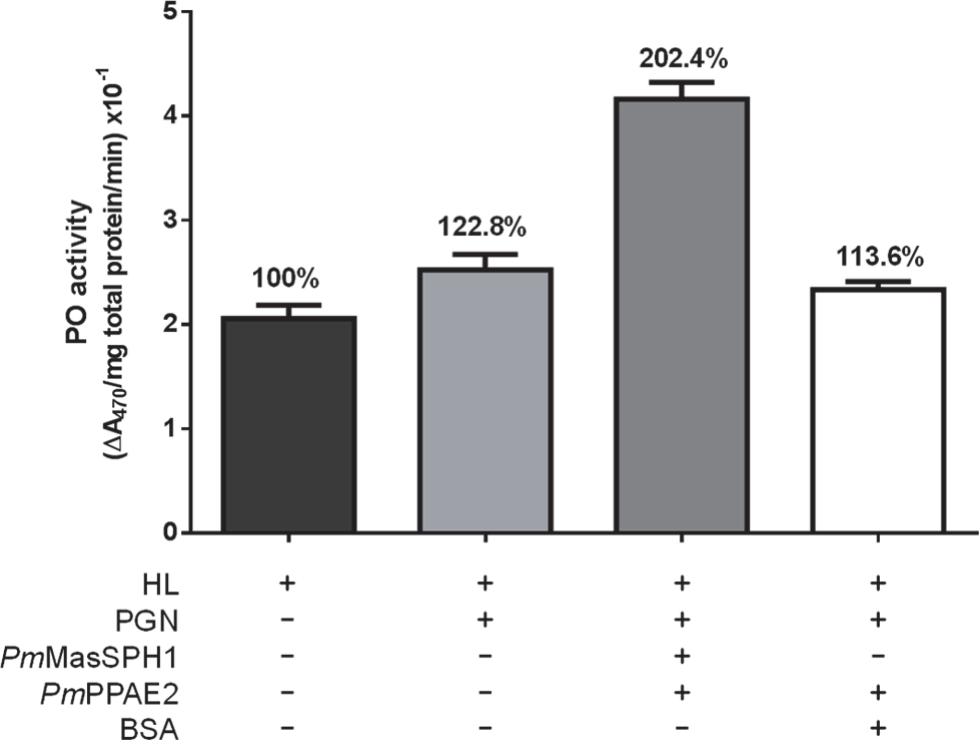
Enhancement of PGN-triggered hemolymph PO activity by r*Pm*MasSPH1 and r*Pm*PPAE2 *in vitro*. The total PO activity of shrimp hemolymph (HL) alone or mixed with *B*. *subtilis* PGN, r*Pm*MasSPH1 and r*Pm*PPAE2 was determined. BSA served as a protein control. Each bar represents the PO activity as the mean ± standard deviation from three replicates. The percentages of each treatment are relative to the PO activity of HL alone.

Because *Pm*MasSPH1 can recognize PGN and bind to *Pm*PPAE2, we examined the role of these proteins in proPO activation. r*Pm*MasSPH1, r*Pm*PPAE2 and PGN were mixed with shrimp hemolymph and then PO activity was measured using L-DOPA as a substrate. In the presence of PGN, shrimp hemolymph PO activity was induced by approximately 23% compared with the non-activated hemolymph (HL) (Fig. 9). Interestingly, high PO activity from PGN was significantly enhanced by 102% when r*Pm*MasSPH1 and r*Pm*PPAE2 was graduated added to the hemolymph (Fig. 9). In addition, incubation of HL with PGN together with *Pm*PPAE2 and BSA (instead of r*Pm*MasSPH1) resulted in no significant change in PO activity (14% compared to the control HL) (Fig. 9). These results suggest that hemolymph PO activity is slightly activated by PGN and *Pm*MasSPH1 may be a co-factor to *Pm*PPAE2 in *P*. *monodon* proPO activation.

## Discussion

In invertebrates, including insects and crustaceans, the proPO-activating system is essential for the melanization process that rapidly responds to the intruding pathogens [4–6,11]. The activation of zymogen proPO to active PO, a critical step required for melanin synthesis, depends on the cleavage of terminal clip-serine proteinase and sometimes occurs with the aid of non-catalytic serine proteinase homologues (SPHs) cofactors [4–6,9]. Several studies have shown the requirement of SPHs for proPO activation by enhancing the cleavage of proPO to active PO by PPAEs [20,23,24]. For example, in insects, proPO activation of *Holotrichia diomphalia* needs PPAF-I to cleave proPO (79 kDa) into PO (76 kDa), and non-catalytic PPAF-II to bind the cleaved proPO and generate the catalytically active PO [23]. The process in *Manduca sexta* requires both PPAE1 (PAP-1) and SPH to simultaneously activate the inactive proPO [20].

In the shrimp *P*. *monodon*, *Pm*MasSPH1 has been previously identified and characterized as a multifunctional immune protein because it possesses opsonic abilities, antimicrobial activity against gram-positive bacteria, and binds to LPS, *V*. *harveyi* and virus [34–36]. Nevertheless, the function of *Pm*MasSPH1 as it relates to proPO activation and other *Pm*MasSPHs remains unknown. Therefore, in the present study, we examined *Pm*MasSPH1 and the newly identified *Pm*MasSPH2 for their potential roles in proPO activation. The pattern recognition properties of *Pm*MasSPHs were also investigated because knowledge on the recognition of PGN by *Pm*MasSPHs has not yet been reported.

The novel *Pm*MasSPH2 consists of an N-terminal clip-domain and a C-terminal SP-like domain with a catalytic triad containing a Gly residue (instead of a Ser residue) similar to what has been previously identified in *Pm*MasSPH1 and other arthropod SPHs. A multiple sequence alignment showed that *Pm*MasSPH2 has a similar primary structure as *Pl*SPH1, *Pl*SPH2, *Hd*PPAFII and *Sp*-SPH, containing a one clip-domain at the N-terminus and an SP-like domain at the C-terminus, unlike *Pm*MasSPH1, which contains a glycine-rich domain at the C-terminus. The highest similarity was found between *Pm*MasSPH1 and *Pl*SPH2 and between *Pm*MasSPH2 and *Pl*SPH1, suggesting that a similar role for *Pl*SPHs in proPO activation.

In the developmental expression profile of *Pm*MasSPHs, we found that both transcripts are expressed in all larval stages tested, similar to other proPO-related genes such as *Pm*PPAE1 and *Pm*proPO2 [31], although *Pm*MasSPH2 had a slightly low level of gene expression at the N3 stage. This is in contrast to *Pm*PPAE2 and *Pm*proPO1 transcripts that are expressed at the middle phase of larval development (e.g., mysis and post-larvae stages) [31,41]. It has previously been suggested that *Pm*PPAE1 and *Pm*proPO2 together might function at an early stage of larval development while *Pm*PPAE2 and *Pm*proPO1 may play a role at later developmental stages [31]. However, it was shown in *P*. *monodon* that there is no PO activity at the N4 stage, suggesting incomplete proPO activation [41]. In addition, the absence of proPO expression in the middle phase of embryo development has also been reported in the crayfish *Pacifastacus leniusculus* [42]. Thus, it is possible that the presence of *Pm*MasSPHs at early larval developmental stages of *P*. *monodon* might function in immune defense reactions other than in proPO activation. This hypothesis is also supported by previous work that shows that *Pm*MasSPH1 has multiple immune functions including opsonic capabilities, antimicrobial activity and recognition of bacterial pathogens [35].

To evaluate the relevance of *Pm*MasSPHs in the proPO-activating system, *in vivo* silencing of *Pm*MasSPH1 and *Pm*MasSPH2 using dsRNA was carried out and total shrimp PO activity was then examined. In *Pm*MasSPH1 and *Pm*MasSPH2 silenced shrimp, the hemolymph PO activity was significantly decreased compared to the control GFP dsRNA by approximately 67% and 64%, respectively. In comparison to the other shrimp proPO-related genes, this decrease in *Pm*MasSPHs is quite high and resembles *Pm*proPO1 and *Pm*proPO2 silencing in which the PO activity was reduced by 75% and 73%, respectively [16]. In addition, knockdown of *Pm*PPAE1 and *Pm*PPAE2 in *P*. *monodon* reduced the hemolymph PO activity only by 37% and 41%, respectively [31]. All evidence suggests that *Pm*MasSPHs, *Pm*PPAEs and *Pm*proPOs together participate in proPO activation, especially *Pm*MasSPHs that are required factors in the activation cascade because their gene silencing resulted in a significant reduction of PO activity. Similarly, in *P*. *leniusculus*, knockdown of *Pl*proPO, *Pl*SPH1 and *Pl*SPH2 also disrupt PO activity, indicating their essential roles in proPO activation [14,28].

Because the proPO system is known to rapidly respond to intruding pathogens and silencing of the *Pm*MasSPHs significantly decreases PO activity, the correlation between the reduction in PO activity by *Pm*MasSPH1 and *Pm*MasSPH2 knockdown and bacterial clearance activity was examined *in vivo*. The number of viable *V*. *harveyi* was significantly increased in the hemolymph of *Pm*MasSPHs silenced shrimp thereby suggested the importance of *Pm*MasSPH1 and *Pm*MasSPH2 in bacterial clearance. It was noted that there are differences in the rate of *V*. *harveyi* clearance between *Pm*MasSPH1 and *Pm*MasSPH2. In comparison to the control GFP dsRNA, the bacterial number in *Pm*MasSPH1 silenced shrimp was higher than in *Pm*MasSPH2 silenced shrimp (11.5-fold versus 7.9-fold, respectively) and may suggest a different way to activate proPO and eliminate the bacteria. In addition to *Pm*MasSPHs, knockdown of *Pm*PPAEs and *Pm*proPOs may also increase bacterial loads and induce high mortality in shrimp [16,30,31]. Likewise, in *P*. *leniusculus*, silencing of *Pl*proPO decreases PO activity and increases hemolymph bacterial number [14]. These relationships may explain the significant role of *Pm*MasSPHs in promoting the PO activity and subsequently limiting the number of bacteria in shrimp hemolymph.

A previous report in shrimp demonstrated that suppression of proPO genes (*Pm*proPO1 and *Pm*proPO2) alters the expression levels of other proPO-related genes and antimicrobial peptide (AMP) genes [15,32]. In the present study, the effect of *Pm*MasSPHs gene silencing on immune gene expression was therefore investigated. We found that transcription levels of the proPO-related gene (*Pm*PPAE2) and AMP genes (*Penmon*PEN3, *Penmon*PEN5, Crustin*Pm*1 and Crus-like*Pm*) were significantly decreased by suppression of *Pm*MasSPH1 but not *Pm*MasSPH2 transcripts. This suggests that proPO and *Pm*MasSPH1 could contribute to the crosstalk between the proPO-activating system and the AMP synthesis pathway in shrimp. This phenomenon is often found in invertebrate immunity because several studies have reported that a single regulator plays a role in more than one pathway. For example, in the mealworm *Tenebrio molitor*, Tm-SPE is a terminal serine proteinase that cleaves pro-spӓtzle in the Toll pathway to produce AMPs and also activates proPO and the cofactor SPH1 in the melanization process [25]. This is consistent with a report in *Drosophila* showing that the melanization reaction depends on activation of the Toll pathway and removal of the serine protease inhibitor Serpin27A [43]. In *M*. *sexta*, the hemolymph proteinase HP6 was also shown to activate PPAE1 (proPAP1) in the proPO system and HP8 in the Toll-like pathway [44].

Because it has not yet been reported for the peptidoglycan recognition proteins (PGRPs) in crustaceans, there have been attempts to elucidate the mechanism of proPO activation by peptidoglycan in these animals. In the crayfish *P*. *leniusculus*, the involvement of *Pl*SPHs in PGN-induced proPO activation has been demonstrated [28]. In the mud crab *Scylla paramamosain*, *Sp*-SPH is also able to activate the PO activity triggered by PGN [29]. Nevertheless, these crayfish and crab SPHs have no PGN binding motif typically found in PGRPs [45]. Therefore, in the present study, we investigated whether *Pm*MasSPHs can recognize PGN similar to our previous study showing that *Pm*MasSPH1 could bind to the bacterial cell wall component, LPS [35]. Our ELISA assay clearly shows the ability of both *Pm*MasSPH1 and *Pm*MasSPH2 to bind PGN. The slightly different *K*_d_ value of *Pm*MasSPH1 (6.51x10^-9^ M) and *Pm*MasSPH2 (5.79x10^-9^ M) to PGN suggests that the binding ability of *Pm*MasSPH1 is stronger than that of *Pm*MasSPH2. Thus, we conclude that *Pm*MasSPH1 recognizes PGN but a different shrimp PRP, *Pm*LGBP, binds to LPS and β-1,3-glucan [32] and subsequently leads to proPO activation. Our results also suggest that aside from the PGRPs, there is another mechanism for PGN-induced immunity in crustaceans probably via other immune related proteins that do not belong to the PGRP family. This was also observed in *P*. *monodon* QM and C-type lectin proteins that did not contain a PGN binding motif but could interact with PGN and induce melanization activity *in vitro* [46]. However, the mechanism on how non-PGRP proteins recognize PGN and activate the proPO system requires further investigation.

According to the AMP synthesis pathway in *D*. *melanogaster*, both Toll and IMD pathways are initially activated by different types of PGN that eventually result in nuclear factor-κB (NF-κB)-dependent AMP synthesis [47]. In addition, the requirement of PGRP has been shown for the activation of Relish, an NF-κB transcription factor family [48]. Because *Pm*MasSPH1 have the ability to bind PGN and suppression of its transcription affects AMP gene expression, this might suggest that in addition to proPO activation, *Pm*MasSPH1 may play a role in the Toll-induced AMP synthesis pathway. In *M*. *sexta*, SPH-3 is not only responsible for the proPO system but is also required for the expression of antimicrobial effector genes [21]. In addition, the silencing of *Pm*MasSPH2 did not affect transcription of other genes and therefore, *Pm*MasSPH2 may play a role only in proPO activation.

To clarify the role of *Pm*MasSPH1 in proPO activation, protein-protein interaction assays were carried out because suppression of *Pm*MasSPH1 altered *Pm*PPAE2 transcript levels. We demonstrated a specific interaction between *Pm*MasSPH1 and *Pm*PPAE2, suggesting that *Pm*MasSPH1 anchors and acts as a cofactor to *Pm*PPAE2 to activate *Pm*proPO1 to active PO. This is supported by an *in vitro* assay showing that the presence of both *Pm*MasSPH1 and *Pm*PPAE2 can promote hemolymph PO activity triggered by PGN. The ability to bind microorganisms and activate proPO might suggest an effective immune response method. When bound to a pathogen, *Pm*MasSPH1 may recruit other components such as *Pm*PPAE2 and *Pm*proPO1 to generate active PO at the site of pathogen infection. It has been shown in *M*. *sexta* that after hemolymph proteinase HP21 activates proPAP2 and 3 [49], these active proteinases, along with SPHs, then produce active PO at the site of infection [19]. Although the crucial role of *Pm*MasSPH1 in proPO-related immunity is extensive described in this study, the role of *Pm*MasSPH2 is still unclear and requires further investigation. In summary, this study is the first to demonstrate that shrimp SPHs function as pattern recognition proteins for Gram-positive bacteria, act as co-activators of proPO and are involved in AMP synthesis.
